# Ten years of research on the sound quality of cochlear implants

**DOI:** 10.3389/fnhum.2026.1818601

**Published:** 2026-07-22

**Authors:** Michael F. Dorman, Sarah C. Natale, Joshua S. Stohl

**Affiliations:** 1 Arizona State University, College of Health Solutions, Tempe, AZ, United States; 2 MED-EL North American Research Laboratory, Durham, NC, United States

**Keywords:** acoustic plus electric hearing, cochlear implant–neuroprosthesis, electrode insertion depth, single-sided deafness, sound quality

## Abstract

This report summarizes data collected over the span of more than a decade from four laboratories on the sound quality of cochlear implants. Sixteen projects (ten published and six unpublished) are summarized; two video and eleven audio files accompany the summaries. Fourteen of the projects used single sided deaf patients (SSD) fit with an implant (CI) as listeners; one project used SSD-CI patients with preserved hearing (HP) in their implanted ear; one project used conventional CI patients. Approximately a third of the SSD-CI patients could create close matches to CI sound quality. The matches varied as a function of electrode length. Patients fit with shorter electrode arrays used upshifted formant frequencies and/or voice pitch and spectral smearing to match sound quality. Approximately half of the patients with longer electrode arrays used low- or band-pass filtering, spectral smearing and/or pitch contour flattening to match sound quality. Other patients chose very few modifications – these signals were relatively clean. For the SSD-CI/HP patients, stimulation directed to both the CI and acoustic hearing was characterized, relative to stimulation directed only to the CI, by one or more of the following: (i) a lower F0, (ii) a less flattened F0 contour, (iii) less upshift in formant frequencies, (iv) less smear, and (v) fewer instances of high-pass filtering. The final project established that muffled sound quality could be reduced by including an input gain for mid frequencies.

## Introduction

In this paper, we review published and unpublished research we have conducted over the span of more than a decade on the sound quality of speech experienced by listeners fit with a cochlear implant (CI). The work was motivated by a question asked by almost all prospective CI patients, “What will my implant sound like?” (see Supplementary material 1: Introduction).

Most of our work has been with single-sided deaf (SSD) listeners fit with a CI (SSD-CI) because these listeners allow us to create a signal for their normal-hearing (NH) ear that potentially matches the sound quality of the signal presented to their CI. Our test procedure has been to present a clean speech signal to the CI *via* a direct-connect cable and then present a modified speech signal to the NH ear *via* an insert earphone or monaural, circum-aural headphone and ask whether that signal matches the sound quality of the signal delivered to the CI. A moderator interacts with the patient to modify the signals to the NH ear to create signals that, if all goes well, closely approximate the sound of the CI (see Supplementary material 2: Video matching procedure). At the end of testing, patients are asked to rate the similarity of the final match to CI sound quality on a scale of 1 to 10 with a score of 10 indicating a complete match to CI sound quality. Over a period of years, we have learned to create signals which, in the best cases, closely match the sound of a patient’s CI. Many of these matches have been included as Supplementary material audio files in our previous publications but now are not retrievable due to changes in computer storage systems. For that reason, we include audio and video files from both old and new projects in this review. Due to convention, the audio and video files are labeled Supplementary files. The files are, in fact, central to this review. Listening is the best way to appreciate the sound quality experienced by CI patients.

## Patients describe CI sound quality

Before describing the results of our matching experiments with SSD-CI patients, we review the results of a project in which conventional CI patients, i.e., patients fit with unilateral, bilateral and bimodal CIs, described CI sound quality (Dorman et al., 2025). Our long-term aim was to assess the relationship between verbal descriptions of sound quality, as might be provided in a clinic, and objective matches to sound quality.

At the German Hearing Center (DHZ), Hanover, Germany, 255 CI patients [89 Advanced Bionics; 86 Cochlear Corporation; 80 MED-EL] filled out a questionnaire about CI sound quality for two time (T) points: (i) shortly after activation (T1) from memory and (ii) at the time of filling out the questionnaire (T2). The mean CI experience at T2 for the three groups ranged from four to eight years. The questionnaire was composed of 25 adjectives describing sound quality. The results at T1 are shown in Table 1.

**Table 1:** Proportion (x100) of patients using a given descriptor for sound quality at T1, i.e., soon after activation.

Sound quality	Boomy	Clear	Computer-like	Distorted	Far-away	Full	Grainy	Grungy	Harsh	High-pitched	Hollow	Metallic	Mickey Mouse-like	Muddy	Muffled	Nasal	Rich	Reverberant	Shrill	Smeared	Smooth	Telephone-like	Thin	Treble-y	Warm
AB	10	20	37	12	18	1	12	17	12	21	8	34	27	16	8	4	2	11	13	2	1	9	8	28	2
Cochlear	15	19	35	21	8	6	15	20	5	12	12	27	34	15	9	2	1	23	15	6	0	9	1	35	0
MED-EL	14	19	31	18	18	6	8	14	8	11	19	23	21	15	9	4	1	16	9	0	1	9	1	29	3

Data from Dorman et al. (2025).

**Figure 1:**
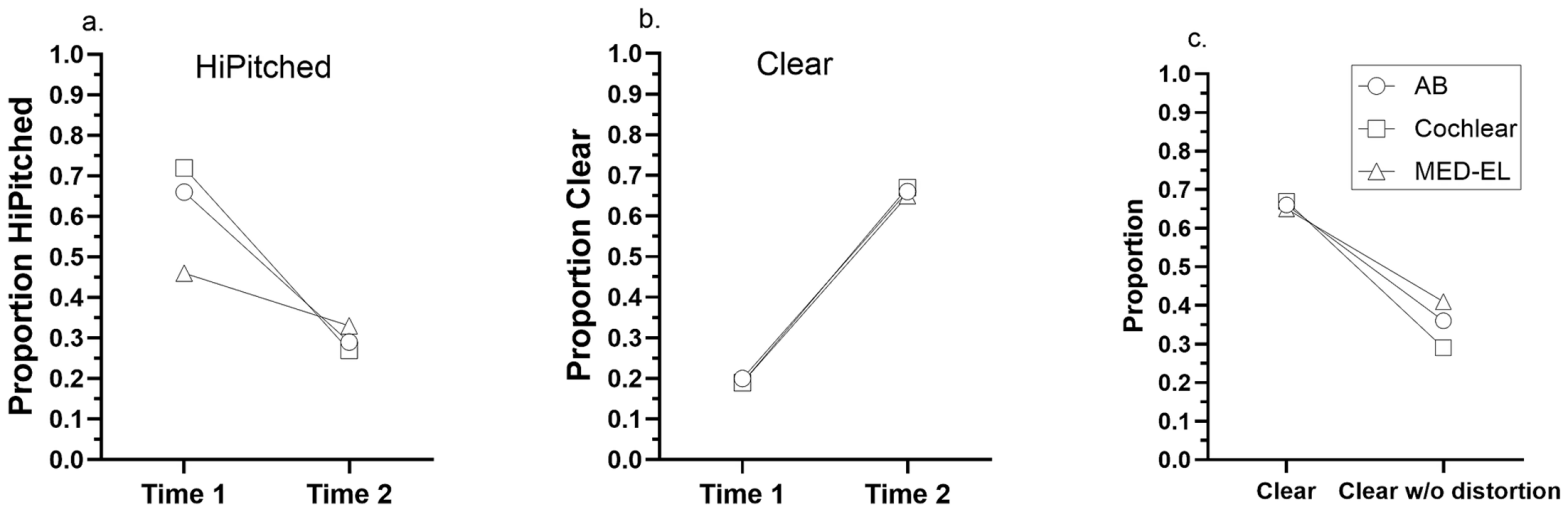
**(a)** Proportion of HiPitched responses at Time 1 and Time 2. **(b)** Proportion of clear responses at Time 1 and Time 2. **(c)** Proportion of Clear and Clear without Distortion responses at Time 2. Figure modified from Dorman et al. (2025). Descriptions of sound quality shortly after device activation and after several years’ experience. With experience Hi-Pitched responses decrease and Clear responses increase.

**Figure 2:**
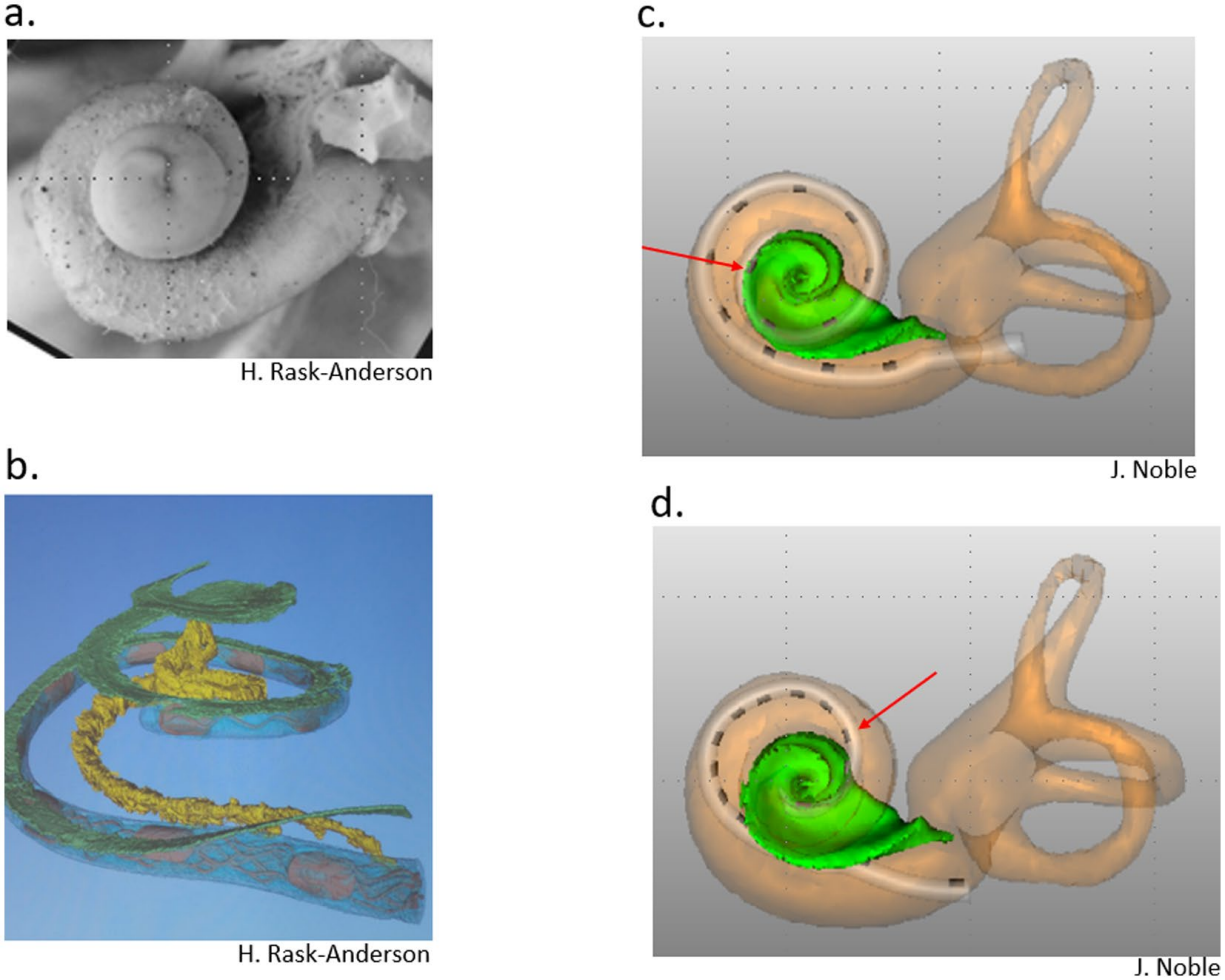
**(a)** Plastic mold of a human cochlea. **(b)** Synchrotron image of a cochlea with the bony matrix digitally removed showing the basilar membrane in green, the spiral ganglion in yellow and an electrode array in blue with individual electrodes in red. **(c)** Computer reconstruction from CT scan of a 28 mm electrode array in a patient. The most apical contact is at the 390 Hz SG frequency. **(d)** Computer reconstruction of 16.5 mm electrode array in a patient. The most apical contact is at the 629 Hz SG frequency. Red arrows point to the most apical electrode in each array. Spiral ganglion is colored green. CT and synchrotron images of longer and shorter electrode arrays in the cochlea.

Just after activation, the most used descriptors were Computer-like, Treble-y, Metallic, and Mickey Mouse-like. The descriptors Treble-y, Metallic and Mickey Mouse-like point to a high-pitched sound quality. For that reason, we combined High-pitched, Metallic, Mickey Mouse-like, Shrill and Treble-y responses into a superordinate category of HiPitched. The results for this category are shown in Figure 1a. Listeners with the longer MED EL electrode array (28 mm) showed significantly fewer HiPitched responses (46%) than listeners fit with the shorter AB 16.5 mm array (66%) or Cochlear arrays (72%). [Cochlear arrays can range from 17 mm to 25 mm (Dhanasingh and Jolly, 2017)]. This outcome is reasonable given the relationship between electrode insertion depth and spiral ganglion (SG) frequency (e.g., Stakhovskaya et al., 2007; Helpard et al., 2021). As shown in Figure 2, the deepest electrode in a 28 mm electrode array stimulates a more apical, or lower frequency, location along the spiral ganglion than the deepest electrode in a 16.5 mm array.

As shown in Table 2, after years of experience, i.e., at T2, the most used descriptors were Clear, Full, Rich and Warm. The most common response was Clear. As shown in Figure 1b, average Clear responses improved from approximately 20 percent shortly after activation to 66 percent after years of experience. To examine further the Clear response, we created a superordinate category of Clear without Distortion. The rationale was that a signal can be clear and yet be abnormal in some manner. For example, both a Mickey Mouse-like voice and a Darth Vader-like voice can be clear but outside the range of normal.

**Table 2:** Proportion (x100) of patients using a given descriptor for sound quality at T2, i.e., after years of experience.

Sound quality	Boomy	Clear	Computer-like	Distorted	Far-away	Full	Grainy	Grungy	Harsh	High-pitched	Hollow	Metallic	Mickey Mouse-like	Muddy	Muffled	Nasal	Rich	Reverberant	Shrill	Smeared	Smooth	Telephone-like	Thin	Treble-y	Warm
AB	7	66	13	6	11	27	6	9	11	19	7	12	8	7	9	2	20	13	0	2	4	4	7	11	10
Cochlear	3	67	14	6	9	19	3	8	9	12	3	10	5	5	3	2	15	8	8	0	6	1	3	7	17
MED-EL	0	65	13	1	10	25	4	9	1	14	5	6	3	8	13	1	8	18	10	1	3	4	3	13	19

Data from Dorman et al. (2025).

Clear responses at T2 were examined and the number of Clear responses without an indication of distortion, e.g., Clear and Warm, were tallied. As shown in Figure 1c, Clear without Distortion was found for 36% of the AB patients; 29% of the Cochlear patients and for 42% of the MED-EL patients. Thus, at T2 only about a third of the patients described sound quality as Clear without Distortion. While not as encouraging as two thirds of the patients responding Clear, we should wonder how it is that even a third of the patients used this category given the very long list of distortions in the auditory signal engendered by electrical stimulation. A partial list of distortions includes (i) a spectral representation that is both sparse, broad and, in many cases, upshifted in frequency (Wilson et al., 2016), (ii) frequency resolution on the order of 10 times poorer than normal (Zeng, 2002), (iii) a reduction in the number of discriminable steps in intensity (Nelson et al., 1996), and (iv) an amplitude/time representation that does not faithfully mimic the amplitude envelope of the signal (Wilson et al., 1997).

After experience with CI stimulation, as shown in Figure 1a, responses in the superordinate category of HiPitched dropped from a high of 72 percent to 30 percent with no difference in outcome among the three groups of patients. We suggested earlier that it was reasonable to find just after activation that patients with shorter electrode arrays would report more HiPitched responses than patients with the longer MED-EL array. This was because the most apical, or deepest, electrode in a shorter array would stimulate more basal locations along the spiral ganglion than the most apical electrode in a longer array. What then accounts for the absence of a difference among the groups after years of experience?

Overall, the results of the survey suggested that, shortly after device activation, listeners fit with different CIs experience different sound qualities for speech. After years of experience with a CI, the data suggests there may not be large differences in speech sound quality among CI listeners fit with different devices. The proportion of Clear responses and the proportion of Clear without Distortion responses did not differ among the three groups of listeners. This outcome, that after years of experience there are not large differences in sound quality experienced by CI listeners, is counter intuitive. If this were the case, then cortical, top-down processes must override both (i) the differences in excitation from spiral ganglion neuron populations stimulated by different length electrode arrays and (ii) the different patterns of electrical stimulation produced by different signal processing and output algorithms. We return to this issue later.

## Sound-quality matching by SSD-CI patients

Descriptions of sound quality, like those reported above, are extremely difficult to verify. If a patient indicates that a signal from a CI sounds Clear without Distortion then, in the absence of convincing data to the contrary, we must accept that the percept is, indeed, clear and is without distortion. In contrast, SSD-CI patients can provide objectively verifiable data about sound quality. As described in the first section of this review, our test procedure has been to present a clean speech signal to the CI and then present a modified speech signal to the NH ear and ask whether that signal matches the sound quality of the signal delivered to the CI. A moderator interacts with the patient to modify the signal to the NH ear to create signals that come progressively closer to the sound of the CI. If the matching signal is constructed with a flattened pitch (intonation) contour, a 30 Hz increase in fundamental frequency (F0) and is low pass filtered at 2 kHz, then we have not verified a Clear without Distortion report. It is in this sense that SSD-CI patients can provide objectively verifiable data about CI sound quality. Before we describe our research using this test procedure, we review several reasons why our efforts could fail.

One reason is that electrical stimulation could produce neural activation patterns so different from the patterns created by acoustic stimulation of the cochlea that acoustic stimulation could not recreate those patterns. Another reason why our efforts could fail is that patients need to be able to ‘hear out’ multiple abnormalities in the CI signal. It seems unlikely that every patient, or even many patients, will have the analytical skill to achieve this. It is also the case that the moderator must be able to take a description from the patient, e.g., “it sounds like someone is speaking with their hand over their mouth” and know which acoustic dimensions to manipulate to achieve that sound quality in a signal directed to the NH ear. All of this is to say that creating a very close match to CI sound quality is far from assured. Indeed, if the first reason for failure listed above is true, then it will not be possible at all.

We turn now to our work with SSD-CI listeners. In 2006, before SSD-CI patients were available for testing in the United States, we tested a MED-EL patient whose pure-tone thresholds in the ear contralateral to the CI were 65–75 dB HL from 250 Hz to 8 kHz (Dorman et al., 2007). The wide range of audible frequencies enabled our initial attempt to match the sound quality of a CI. The patient’s verbal description suggested that the CI was missing both low and high frequencies. A bandpass filtered, sine-vocoder signal produced the best, but not very convincing, match. The magnitude of the hearing loss in the better ear confounded an interpretation of this result.

## First patients

In 2014, we began sound quality matching experiments using SSD-CI patients with normal hearing-thresholds in the ear contralateral to their CI [Svirsky et al., 2013 has the first publication in this field]. The patients were from a cohort implanted by Daniel Zeitler, M. D. Our first patient had been followed for several years in a study of cortical plasticity (Sharma et al., 2016). The patient was born with normal hearing bilaterally and began to lose hearing in one ear at age 5. At age ten, that ear received a MED-EL CI.

Before implantation, cortical evoked-potentials revealed an altered cortex due to unilateral deafness. Approximately three years after device activation, the patient was asked to match the sound quality of her implant. At this point in time, the deafness induced cortical reorganization had partially disappeared. Thus, the patient was perceiving speech using a twice reorganized cortex – reorganized first by unilateral auditory deprivation and then reorganized again by unilateral CI stimulation. Using a primitive version of the interactive procedure described earlier, CI sound quality was matched to a signal that was bandpass filtered between 0.4 kHz and 1.0 kHz and had been flanged – a delay and add back operation with a slowly varying delay over time that produces a phasing quality. The match, and the patient’s confidence in that match, can be heard in Supplementary material 3: Video of first patient’s match. As shown in the video, the patient dismissed both sine and noise vocoders (see below) peremptorily. Critically, the patient commented that her CI sounded like someone was talking from behind a door or was talking with a hand over their mouth. This observation was consistent with her match to a bandpass filtered signal and was consistent with the report of the patient tested in 2006.

The next step was to assess whether other SSD-CI patients would (i) reject the sound quality of vocoders and (ii) choose, instead, a bandpass-filtered, natural-speech signal to make a match to CI sound quality. Here a digression is necessary to explain channel vocoders to provide a context for the use of vocoded signals in our experiments.

## Vocoders

Channel vocoders (or voice encoders) were an early tool for speech synthesis. Vocoders were used to study different implementations of speech transmission systems for telephones (Dudley, 1939; Flanagan, 1972) and played a major role in secure voice communications in World War II (Vocoder, 2025). In a common implementation, the speech signal was divided into *n* continuous frequency bands, the energy in each band was estimated and a signal was output proportional to the summed energy in each filter band. The excitation signal was noise, and the output was noise bands with each noise-band the width of an input filter. Energy estimates from the signal were used to modulate the corresponding noise band carriers, after which bands could be summed to produce a wideband reconstruction of the signal. Using this tool, researchers could, for example, determine the minimum number of bands necessary to transmit speech with a high degree of intelligibility via a telephone transmission line.

As should be expected, a noise vocoder has a whispered or noisy voice quality. This was not an issue for these experiments as the goal was to study intelligibility. An alternative output could be generated by using a set of sine waves each at the center frequency of a filter band. This produces a tonal voice quality. Noise and sine vocoder outputs can be heard in Supplementary material 4: Audio files for matching, at the end of the file.

Loizou (1998, 2006) noted the similarity in signal processing for channel vocoders and for CIs. For both, the input signal is divided into *n* frequency bands, the energy in each band is estimated and a signal proportional to the summed energy in the filter bands is output. For the CI, rather than noise band carriers, the signal is delivered using bi-phasic pluses *via* electrodes in the scala tympani to multiple, fixed-frequency regions along the spiral ganglion. The number of frequency regions is determined by the number of electrodes and, for some designs, by current steering between electrodes. The frequencies of the stimulated regions are a function of electrode insertion depth, i.e., the distance of the electrodes from the round window and the spread of current.

The similarity in signal processing for cochlear implants and for channel vocoders led to a large number of studies using vocoders to study aspects of signal processing relevant to the design of cochlear implants (for a review see, Cychoaz et al., 2024). For example, several groups investigated the minimum number of channels needed to achieve a high level of speech understanding (e.g., Shannon et al., 1995; Dorman et al., 1997; Loizou et al., 1999). In many experiments, speech understanding outcomes were similar for vocoder simulations of CIs and from studies of patients fit with CIs. This validated the use of vocoders to study speech processing algorithms for CIs. Although it was never claimed that the output of a noise or sine vocoder sounded like a CI, *in the absence of other models*, *vocoders have been used for demonstrations when patients ask, ‘What does an implant sound like*?’

## Vocoded signals or natural speech signals?

**Figure 3:**
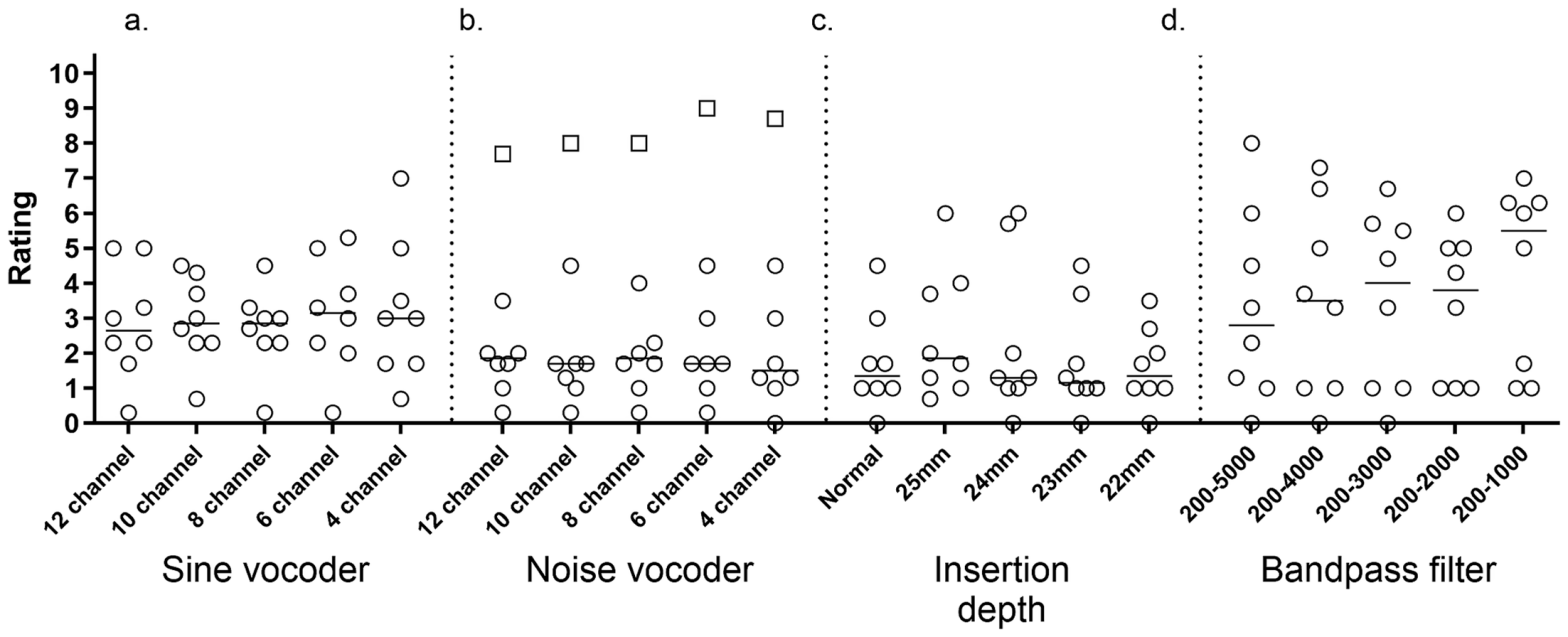
Similarity in sound quality to that of a CI for (i) sine and noise vocoder processed signals as a function of number of channels **(a,b)**, (ii) sine vocoder signals as a function of insertion depth **(c)** and (iii) natural voice signals as a function of bandpass filter frequencies **(d)**. Horizontal bars indicate the median value for each condition. Figure reproduced with permission from Dorman et al. (2017). Similarity of sine vocoders, noise vocoders and filtered natural speech to cochlear implant sound quality.

To gain an initial understanding of the similarity of vocoder outputs to CI sound quality, Dorman et al. (2017) created four types of signals for presentation to the normal hearing ear of eight SSD-CI patients: Noise and sine vocoded sentences with the number of channels as the parameter; sine vocoded sentences with insertion depth as the parameter and natural speech signals with bandwidth as the parameter. Listeners rated the similarity of each of these signals to unmodified signals sent to the CI on a scale of 0 to 10 with 10 being a complete match to the CI signal. The results are shown in Figure 3. Apart from one patient who gave high match ratings to four and six channel vocoder signals, it was clear from the results shown in Figure 3 that the sound quality of the noise and sine vocoders we implemented did not correspond closely to the sound quality of cochlear implants. Other studies with vocoders using different excitation functions have reached a broadly similar conclusion (Peters et al., 2018; Karoui et al., 2019; Svirsky et al., 2021). That said, if the initial stages of signal processing for CIs are like that of a channel vocoder, then why do vocoders not have the same sound quality as an implant?

In a second experiment, we used natural speech signals with three young SSD-CI listeners and found that a combination of bandpass filtering and spectral smearing led to similarity rating scores of 10. These maximum similarity scores were very surprising and it is likely that the young listeners did not use the same internal similarity scale as adults — that is, they may not have been as critical. Nonetheless, once again bandpass filtering was useful to create signals that approximated the sound quality of a CI. We concluded that “natural speech signals that have been muffled to one degree or another by band pass filtering and/or spectral smearing provide a close, but incomplete, match to CI sound quality for many patients.”

## Experiments using natural speech signals

By 2016, to improve our matches, we had added more operations to our signal processing toolbox. Using the STRAIGHT synthesis algorithm (Kawahara et al., 2001) and standard signal processing tools, the signals to the NH ear could be altered by (i) filtering, (ii) spectral smearing, (iii) changing the fundamental frequency (F0), (iv) flattening the F0 contour (or intonation contour), (v) changing formant frequencies, (vi) altering resonances and ring times to create a metallic sound quality, (vii) flanging, (viii) using a noise vocoder or (ix) using a sine vocoder. The operations could be used singly or in any combination. An audio example for each operation can be heard in Supplementary material 4: Audio operations for matching. Most operations are self-explanatory, e.g., a change in F0. However, spectral smearing could be implemented in many ways. Our implementation, modeled after Baer and Moore (1993), broadened formant peaks causing a reduction in the difference in amplitude between spectral peaks and valleys for voiced signals. Because formant amplitudes normally decrease in the series F1, F2, F3, the additional broadening from smearing effectively creates a low-passed signal. At moderate to high values of smear, the signal also has a buzzy quality.

In 2016, with the assistance of the staff at the World Hearing Centre, Institute of Physiology and Pathology of Hearing, Kajetany, Poland, we tested nine SSD-CI patients fit with MED-EL devices. Three of the nine rated their matches as 9.5 on our 10-point scale, i.e., the matches were very close to the sound quality of the CIs. The common dimension for these three matches was low-pass filtering at 1.0 or 2.0 kHz (Supplementary material 5: Match in Polish).

In the larger data set, six of the nine listeners asked for low-pass filtering, band-pass filtering or spectral smearing and eight of the nine needed multiple changes to a clean signal to match CI sound quality. These data indicated that obtaining a good match to CI sound quality was going to involve more signal modifications than the simple band-pass filtering and/or smearing operations requested by our first patients.

One patient, with a very short electrode insertion (320-degree angle) with three electrodes outside the round window and one at the round window, provided a glimpse into the effect of a shorter electrode insertion on CI sound quality. The match (rated 9.5) was shifted up in frequency—as heard in Supplementary material 6: Polish match, short electrode. This suggested that patients fit with fully inserted but relatively short electrode arrays might create matches to CI sound quality using upshifted pitch or formant frequencies. We return to this later.

At this point in our research program, we had data from a small number of English-speaking and Polish-speaking patients that converged on band-pass filtering and/or spectral smearing as commonly used operations to match CI sound quality. These data suggested a muffled percept. However, more patients needed to be tested to confirm or reject this suggestion.

**Figure 4:**
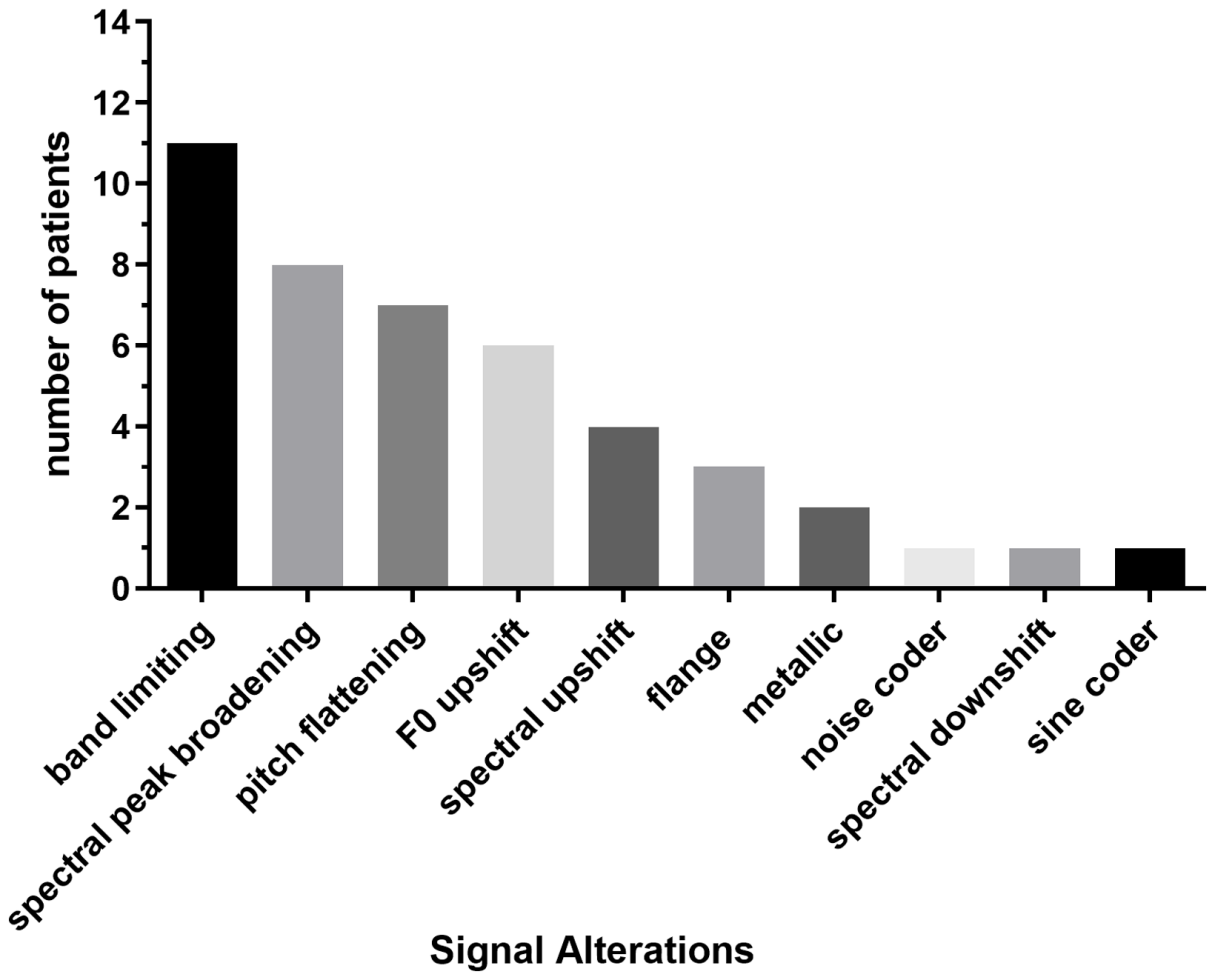
Signal alterations needed to match the sound quality of a CI for 14 patients. Figure reproduced from Dorman et al. (2020). Signal alterations needed to match the sound quality of a CI for 14 patients. The most common alterations were band limiting, spectral peak broadening and pitch flattening.

We described the sound-quality matching results for fourteen, English speaking, SSD-CI patients in Dorman et al. (2020). As shown in Figure 4, the most common alterations to a clean signal were band-pass or low-pass filtering, spectral peak smearing and F0 contour flattening. The data confirmed our speculation that a muffled percept was common. Yet, because, on average, 3.4 operations were used to create a match, the percept was more complex than just muffled. For the fourteen patients, the mean match score was 8.8 with over half of the matches at 9.0 or higher. These data indicated that we could come close to the sound of the CI using the signal processing operations described previously. We examined *very* close matches in Dorman et al. (2024).

**Figure 5:**
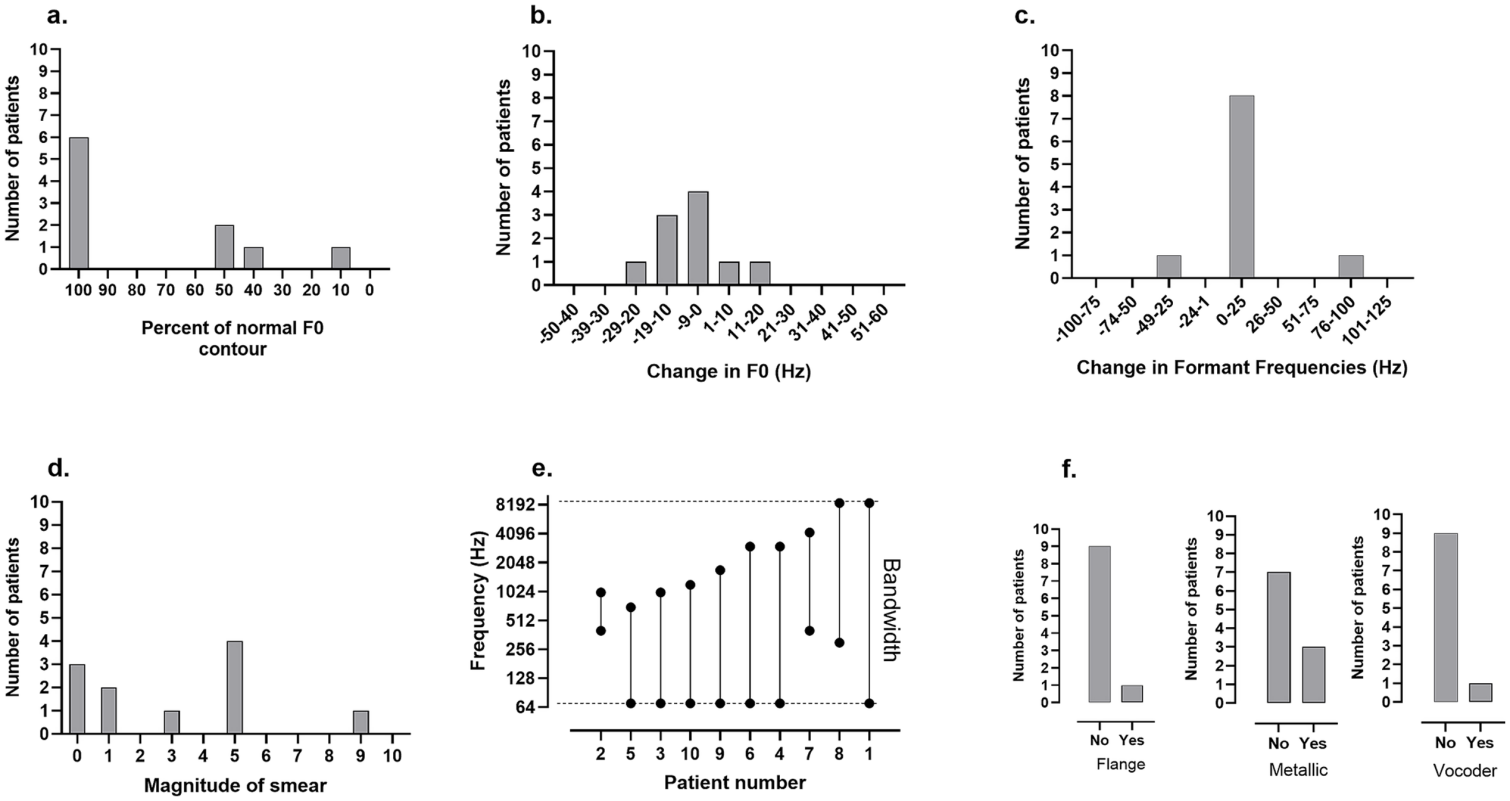
Signal alterations needed to match CI sound quality for 10 patients who rated their matches as 9.5 or higher. The alterations included changes to **(a)** the F0 contour, **(b)** to F0, **(c)** to formant frequencies, **(d)** to Smear and **(e)** to signal bandwidth. Other alterations included **(f)** Flanging, creating a metallic sound quality and use of a vocoder. Figure reproduced from Dorman et al. (2024).

Ten patients were identified from a sample of 30 MED-EL SSD-CI patients using the criterion of a match score of 9.5 or higher. The stimulus manipulations needed to achieve these high match scores are shown in Figure 5. Very few patients needed the flange, metallicize, vocoder, or formant shift operations to match CI sound quality. Flattening of the F0 contour was requested by half of the patients. The flattening ranged from 90% normal to 10% normal. The most common modifications were filtering, spectral smearing and a change in F0.

In this study, two response patterns were identified. Five of the patients asked for a narrow bandwidth—0.6 to 1.6 kHz—and four of these asked for at least moderate smear. An additional patient asked for a narrow bandwidth (0.6 kHz) but no smear. The stimulus dimensions picked by these patients are consistent with a muffled sound quality. In contrast, four of the patients chose either no filtering or at least a 2.9 kHz bandwidth and no or minimal smear to create a match. An additional patient asked for an 8 kHz bandwidth and moderate smear. The minimal or absent band limiting and smear suggests a percept that was less muffled or relatively clean. Audio files for all matches are found in Supplementary material 7: Med-EL matches.

Many, but not all, listeners created matches with muffled sound quality. It is easy to imagine that factors like current spread and the vocoder-like processing used for CIs lead to a muffled sound quality. It is less easy to imagine why patients do not hear this quality. Top-down mechanisms likely play a major role in this outcome, but we do not have converging evidence that this is the case.

It would be reasonable to suspect that the factor underlying a narrow perceptual bandwidth, i.e., needing a bandpass filter to make a sound quality match, is a compressed range of electrode pitches. Previous research suggests that, for many patients, electrode pitch decreases after experience with a CI (e.g., McDermott et al., 2009; Reiss et al., 2007) and comes to approximate input-filter center-frequencies rather than spiral ganglion frequencies (e.g., Tan et al., 2017; see however, Hatley et al., 2025). A general characteristic of this work is that pitch matches for mid- and high-frequency signals are lower than the input frequency. However, individual differences are large and the proportion of patients in the CI population who match to frequencies much lower than the spiral ganglion frequency is not known.

To explore this account, a patient who matched CI sound quality to a signal that was band pass filtered between 0.4 kHz and 1.0 kHz was asked to match the pitch of high-rate pulse trains presented to electrodes E1, E4, E7 and E10 (E1 was the most apical electrode) using sine signals presented to the NH ear. Stimulation at E1 was matched to 110 Hz while stimulation at E10 was matched to 3,575 Hz. Thus, the patient’s sound quality match using a very narrow acoustic bandwidth was not the result of an equally narrow range of electrode pitches. Given this outcome, it is possible that the perceptual effect of a narrow bandwidth in the NH ear mimics the perceptual effect of other abnormalities in signal processing that result from electrical stimulation of the auditory pathway from the CI ear.

## The ideal patient?

When we began this project, we imagined that the ideal SSD-CI patient would be a professional sound engineer in the music industry who had years of experience listening to, and altering, voice signals. We reasoned that this set of skills would enable her to listen with the analytic acumen necessary to create a trustworthy match to the sound of her CI. It would be useful if, in addition, the patient had a recording studio at home where she could devote hours to creating a match. We have tested such a patient.

The patient was implanted with a MED-EL 31.5 mm electrode array. The insertion was very deep – a 777⁰ insertion angle. After extensive interaction with the patient and using signal modification software (REAPER, Cockos, Inc.) designed for music, a match score of 10 out of 10 was achieved. This was achieved for both a male voice and two female voices using the same set of parameters. The matches can be heard in Supplementary material 8: Ideal patient. The modifications needed to make the matches to the male and female voices included a − 10 Hz shift in F0, a + 9 dB gain at 120 Hz, moderate smear, amplitude compression, and a ‘drone’ (a constant frequency tone present throughout the signal) at 174 Hz. Headphones or loudspeakers with very good low-frequency response (to 100 Hz) are necessary to hear the low-frequency drone. Three new dimensions were present in this match (i) frequency specific gain (or EQ), (ii) amplitude compression and (iii) a constant frequency ‘drone’. These dimensions are likely not in the vocabulary of non-musicians/sound engineers and may account for the often-offered comment, ‘I cannot describe what is missing from my match.”

## How close are 9.5 matches to CI sound quality?

In our 2024 paper reviewed above, we described matches to CI sound quality which were rated between 9.5 and 10. Because of these high ratings, it was reasonable to suppose that the sound of the matches came very close to the sound of patients’ CIs. However, we cannot be sure because we cannot hear what a patient’s CI sounds like.

To provide data on this issue, we turned to listeners with normal hearing bilaterally. We designated one ear as the ‘CI ear’ and the other as the NH ear. We directed signals to the ‘CI ear’ that had the properties of matches to CI sound quality, e.g., high pass filtered at 300 Hz, low pass filtered at 2500 Hz and a − 10 Hz shift in F0. We then asked the listeners to create a match in the NH ear to that signal using the same procedures as when testing an SSD-CI patient (the examiner was blind to the characteristics of the target in the ‘CI ear’). The NH listeners were asked to rate the similarity of the match to the target in the same way as the SSD-CI patients rated their matches. Ten targets were created and two NH listeners created matches for each target.

In this test environment, we can hear the target, i.e., the ‘CI’, and can judge how similar a 9.5 or 10 match is to the target. Nine ‘CI’ targets and matches to those targets rated 9.5 and higher can be heard in Supplementary material 9: Typical Hearing matches. In these files the target is presented first and then the match. To our listening, the sound of most matches is close to the sound of the targets. Given this outcome, it is likely that matches rated 9.5 and greater by SSD-CI patients are good approximations to CI sound quality.

## Sound quality of devices with shorter electrode arrays

**Figure 6:**
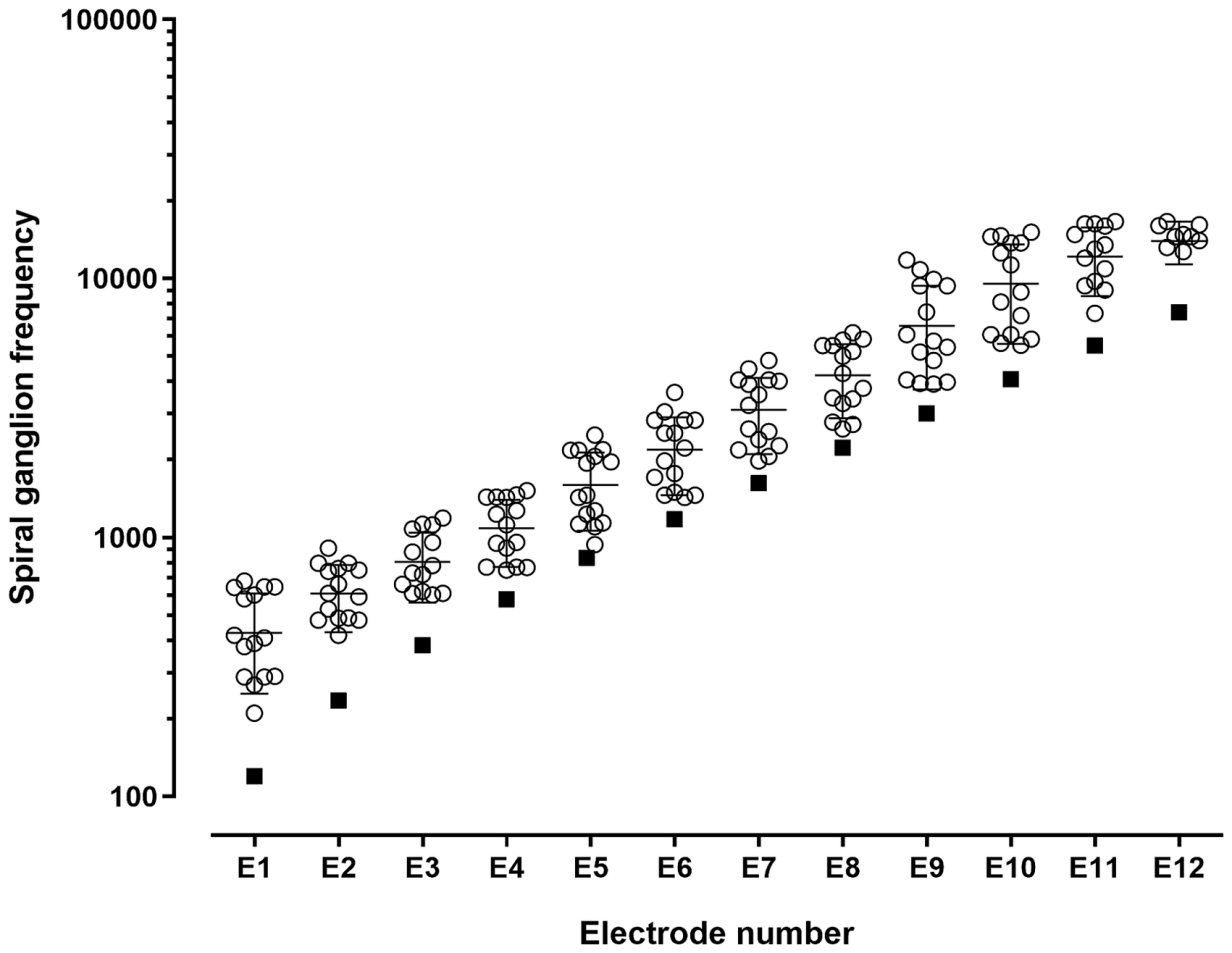
Estimated spiral ganglion (SG) frequencies as a function of electrode number for patients fit with a 28 mm electrode array. Filled squares indicate the filter center frequency for each electrode. Open circles indicate SG frequencies for each listener for stimulation of E1–E12. Horizontal bars indicate mean and standard deviation for each condition. Figure reproduced from Dorman et al. (2020). For patients fit with a 28 mm electrode array, the place of stimulation in the spiral ganglion is always higher than the center frequency of the electrode filter.

In the next section we turn to CI sound quality for patients fit with electrode arrays that are shorter than the 28 mm and 31.5 mm arrays used by the patients described above. Before we describe these results, it is important to note that, even with 28 mm electrode arrays, the places of spiral ganglion stimulation are upshifted with respect to the input filter frequencies (see Figure 6). Yet few patients with this length insertion have asked for an upshift in formant frequencies to match CI sound quality. These data suggest that cortical processes underlying voice recognition tolerate mismatches between input frequencies and spiral ganglion place of stimulation. Will this also be the case for listeners with shorter electrode arrays and larger mismatches?

The listeners in question were SSD-CI patients fit with the Advanced Bionics 16.5 mm electrode array. In the survey data reported in the first section of this review, shortly after device activation patients fit with AB devices reported the superordinate descriptor HiPitched more often than MED-EL patients. We commented that this was a reasonable outcome given the upshift in characteristic frequency of spiral-ganglion neurons stimulated by the shorter electrode array. However, after years of experience with the AB device, patients did not employ significantly more HiPitched descriptors than MED-EL patients. We explored these outcomes in a series of small studies.

The sample in our first test of AB patients was composed of three SSD-CI patients (Dorman et al., 2017). All had six months or less of CI experience at the time of testing. Given the results of the survey data for the period shortly after device activation, we should expect that the listeners would create a match to CI sound quality using signals with a salient high frequency component. This was, indeed, the case. All three patients asked for metallic sound-quality and all asked for upshifts in formant frequencies.

A second study with AB patients employed six SSD-CI listeners (Dorman et al., 2022). The mean SG frequency stimulated by the most apical electrode (E1) was estimated to be 728 Hz with a range from 620 Hz to 890 Hz. In contrast, the mean SG frequency at E1 for patients fit with the longer 28 mm MED EL electrode was 410 Hz with a range from 210 to 680 Hz (Dorman et al., 2020). Thus, the cortical, speech processing routines for AB patients likely received a signal more upshifted in frequency than the cortical speech processing routines for MED EL patients.

Data was collected at two time-points, i.e., with some CI experience (mean = 8 months) and then after several years of CI experience (mean = 36 months). At the first time-point, five of the six listeners asked for an upshift in formant frequencies to match the sound of their CI. The upshifts ranged from 80 to 320 Hz. Four listeners asked for spectral smearing and four asked for an upshift in voice pitch (F0) ranging from 10 Hz to 80 Hz. This outcome was consistent with our survey data.

At the second time-point, i.e., after several years of experience, a similar pattern of results was obtained with five of the six patients asking for an upshifted formant pattern (100–500 Hz), four asking for spectral smearing and four asking for an upshift in F0 (10–80 Hz). Three patients provided sound quality matches of 9.5 or greater. These matches are found in Supplementary material 10: AB matches.

The upshifted matches described in the previous paragraph are not consistent with the survey data. This outcome suggests that the large decrease in HiPitched responses and responses indicating Clear without distortion in the survey data after years of CI experience should not be interpreted to mean that the underlying representation of the signal has been normalized. or, at least, not normalized for all patients.

In the study described above, there could be large differences between descriptions of sound quality and matches to sound quality. For one patient, the description of sound quality included the terms clear, crisp., detailed, dynamic, full, lush, normal and rich. However, the match (rated 9.8) was composed of F0 = +80 Hz; F0 contour = 25% normal; formant = +100 Hz; smear = high; bandwidth = HP 400 Hz; and a metallic sound quality. This signal was far from normal in multiple dimensions. Other patients also used the descriptor ‘normal’ but matched to signals with upshifted F0 and/or formant frequencies, high degrees of smear, or a flattened F0 contour. We propose a mechanism for the divergent outcomes from survey descriptions of sound quality and from our matching procedure later in this review.

## Matches from long and short electrode patients

The difference in sound quality for 9.5 and higher matches for experienced patients fit with longer and shorter electrode arrays support the view that, at some level, the internal representation of speech for long and short electrode patients, after years of experience with their CI, are different despite similar responses on our survey of descriptors for CI sound quality. The strongest test of this hypothesis would use a within-subjects comparison rather than a between-subjects comparison because AB and MED-EL devices differ in signal processing as well as in the length of electrode array. Fortunately, MED-EL electrodes can be functionally shortened by turning off the most apical channels and reassigning the apical filter bands to more basal channels. AB electrodes can be functionally lengthened by creating a current steered, virtual electrode (Phantom electrode) more apical than the most apical physical electrode (Saoji and Litvak, 2010). In this fashion, a within-subjects comparison of longer or shorter electrode arrays can be made while holding signal processing constant.

Five SSD-CI patients with MED-EL devices and two with AB devices participated in this project (Dorman et al., 2019). For the MED-EL patients, when the electrode array was functionally shortened, the matches to CI sound quality were characterized by upshifts in the formant pattern up to 150 Hz or by increases in F0 up to 20 Hz (see Supplementary material 11: E1/E2 off). For the AB patients, matches in the lengthened electrode condition contained formant frequencies approximately 100 Hz lower than in the baseline condition. Perceptually, however, the differences were very small. Taken together, these results remove the confound of electrode length with signal processing strategies and are consistent with the view that electrode length is a significant factor in determining CI sound quality.

## Rationalizing the sound quality of CIs with shorter electrode arrays

On average, the human vocal tract is longer for a male than for a female. The longer vocal tract results in lower resonant, or formant, frequencies for men than for women or children (Fant, 1970; Peterson, 1952). For that reason, the location of formant frequencies informs a listener about the vocal tract length, and size, of the talker (Smith and Patterson, 2005; Fitch, 1999). If male-voice formant frequencies are delivered to abnormally high SG frequencies due to a short electrode insertion, then the elevated formant frequencies may specify a female vocal tract, instead of a male vocal tract. In this circumstance, cortical voice-recognition systems would be presented formant frequencies from a male talker residing in the frequency domain of a female talker or even a child talker. These upshifted and discordant pieces of information most likely contribute to the unusual voice quality heard in Supplementary material 4: Audio for formant shift = 200 Hz.

Voice pitch (F0) can also be altered if a signal is delivered to abnormally high SG frequencies. Many studies have documented increases in pitch/timbre when a fixed-frequency signal is delivered to increasingly basal electrode locations [e.g., Eddington et al., 1978; for constraints on this outcome see Adel et al., 2019; Marozeau et al., 2020]. Together, the alterations to the formant pattern and voice pitch, coincident with delivering a male voice signal to inappropriately high SG frequencies, are very likely to be major factors in the unusual voice quality heard in Supplementary material 10.

## SSD-CI patients with preserved hearing

Now we turn to another group of SSD patients—those with preserved hearing (HP) in the implanted ear (SSD-CI/HP). These patients are of interest because they can make sound quality matches for signals directed to their CI, their acoustic hearing and to both CI and acoustic hearing. Once again, we turned to our colleagues at the World Hearing Center, Kajetany, Poland for assistance. Four patients were tested at the World Hearing Center; an additional three were tested in the United States.

For SSD-CI/HP patients, most surgeons insert an electrode array up to, but not overlapping, the frequency domain of acoustic hearing, or leave a gap between the frequency domains of the electric and acoustic stimulation (see Gifford et al., 2017). These insertions can effectively create a short electrode array – the length of which depends on the upper frequency extent of acoustic hearing. Channel programming can differ from that for a conventional CI with upshifted, filter-center-frequency to electrode assignments.

When stimulation was directed to the CI alone, matches to sound quality were characterized by one or several of the following: (i) increases in F0 or formant frequency, (ii) reduced extent of the F0 contour, (iii) low-pass or band-pass filtering and (iv) a moderate to high degree of spectral smear (creating a very muffled sound quality). The latter confirms that muffling is not exclusive to patients with longer electrode insertions.

Stimulation directed to the region of preserved hearing was matched to a signal created with a low-pass filter similar in high frequency cut-off to the upper frequency extent of acoustic hearing. For several patients in this condition, extra sounds were heard. One patient described these sounds as similar to a ‘brush on a drum’. It is probably the case that a simple low-pass filter does not capture all the sound quality of stimulation directed to the region of acoustic hearing.

Stimulation directed to both the CI and acoustic hearing was characterized, relative to stimulation directed only to the CI, by one or more of the following: (i) a lower F0, (ii) a less flattened F0 contour, (iii) less upshift in formant frequencies, (iv) less smear and (v) fewer instances of high-pass filtering. These effects are reasonable given the added F0 information from the region of acoustic hearing. For some patients, this resulted in better sound quality. Audio examples of matches in the three test conditions, i.e., CI-only, acoustic hearing only and combined CI and acoustic hearing, can be found in Supplementary material 12: E, A and E plus A.

## An extreme outlier

As heard in the Supplementary material audio files, there is significant within-group variability in sound quality for patients fit with longer and shorter electrode arrays. Here we describe the most extreme outlier from the sample of patients with shorter arrays. The patient was from our Polish sample of SSD-CI patients with hearing preservation. Because the patient had preserved hearing to 1 kHz (</= 20 dB HL to 750 Hz; 60 dB HL at 1 kHz), the electrode insertion was very short, i.e., an insertion angle of 289 degrees and a SG place frequency of 1,357 Hz. The patient was programmed as if the electrode array were fully inserted with fine structure processing implemented for the four most apical electrodes. Given the short insertion, there was an extreme mismatch between filter frequencies and SG frequencies, e.g., the most apical electrode’s filter CF was 291 Hz and the SG frequency was 1,357 Hz. It was reasonable to guess that this patient, in the electric-stimulation-only condition, would create a match to CI sound quality with very upshifted formant frequencies and F0. However, the match, rated a 9.0, was crafted with an F0 increase of only 15 Hz, a low-pass filter at 2.0 kHz and a moderate amount of smear. The patient noted that the CI signal had a slightly metallic sound quality. This outcome demonstrates without question that there are mechanisms that can compensate for, or normalize, large abnormalities in the relationship between input frequencies and place of stimulation in the SG.

## Questionnaire data *vs*. matching data

Throughout this review we have pointed out instances in which data from stimulus matching differs substantially from data collected by questionnaires or oral reports. In these instances, after years of experience with a device, the questionnaire data suggests a relatively clear signal while the matching data suggests a degraded signal. How can we account for these very different outcomes?

Dorman et al. (2025) proposed that, after experience with a CI, the decrease in proportion of patients reporting an abnormal signal in the questionnaire data reflects changes in the *salience* of abnormal aspects of the signal rather than changes in the *presence* of abnormal aspects in the internal representation of the signal. In turn, this change may be related to the processing of the signal as familiar *vs*. unfamiliar.

Familiar and unfamiliar voices elicit different patterns of connectivity among voice recognition regions in the anterior and posterior, right, superior temporal sulcus (STS) (von Kreigstein and Giraud, 2004; von Kreigstein et al., 2010), i.e., familiar and unfamiliar voices receive different processing. It is reasonable to suppose that the sound of a CI shortly after device activation is unfamiliar but after years of listening experience becomes familiar. On our view, one effect of processing a signal as familiar is illustrated by the performance of the SSD-CI patient fit with a shorter electrode array described above who, after 3 years of experience, described CI sound quality, via a questionnaire, as clear, crisp., detailed, dynamic, full, lush, normal and rich. However, to match the sound quality of the CI, the signal to the NH ear needed to be altered in multiple dimensions. This outcome indicates that, after years of listening experience and of processing the signal as familiar, abnormalities can be present in the internal representation of the signal but not salient in everyday listening. On this account, both the questionnaire data and the matching data are correct. The different outcomes reflect different levels of stimulus representation accessed by the two test instruments.

## Matches to music

Music plays a significant role in CI patients’ quality of life (e.g., Karaman Demirel et al., 2025). In 2024, we began a pilot project to determine whether patients could create close matches to the sound quality of music. Because our signal modification software was designed for speech, we switched to sound modification software for music, i.e., REAPER (Cockos, Inc.). One musically trained patient created a match rated 9.5 to a vocal and instrumental piece. The patient was familiar with the piece and had used his CI for 10 years. The original and the match, as well as a match to speech rated 9.7, are in Supplementary material 13: Speech and Music. We include this match to highlight the very wide gap in sound quality for speech and music.

## Bench to bedside

In the final project of our research program, we aimed to improve CI sound quality creating signal processing to reduce the muffled percept we have found for many patients. This percept is associated with (i) low-pass or band-pass filtering and (ii) high degrees of smearing for the matching signal in the NH ear. One interpretation of this outcome is that many SSD-CI patients do not have adequate access to mid- or high-frequency components of the signal. If this is the case, then a simple increase in mid-frequency energy may reduce the muffled percept.

In a test with 18 MED-EL CI listeners (4 unilateral, 11 bilateral, 3 SSD-CI), we compared the effects of five input-gain conditions on ratings of degree of muffling for a male voice: (i) no gain, (ii) 4.6 dB, (iii) 9 dB, (iv) 14 dB and (v) 18.5 dB. These gains were measured at 3 kHz. All signals were created off-line and input via a direct input cable.

**Figure 7:**
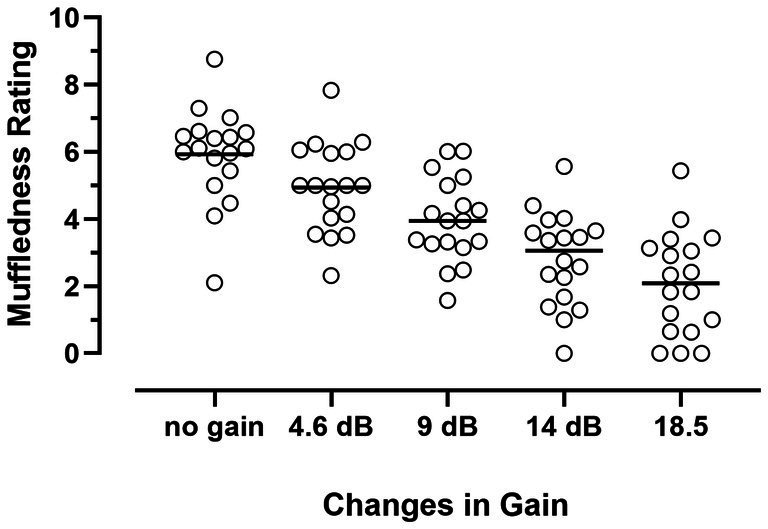
Ratings of degree of muffling for male speech in quiet (*n* = 18). Individual patient performance is indicated by open circles. Mean scores indicated by horizontal line. Lower ratings indicate less muffling. Perception of a cochlear implant signal as muffled decreases as mid-frequency energy is increased.

Patient rating of muffling, using a visual analogue scale ranging from 0 (no muffling) to10 (extreme muffling) in the five test conditions, is shown in Figure 7. All conditions in which mid-frequency gains were applied led to significantly lower ratings of muffling than in the no gain condition. The ratings were: no gain = 5.93; 4.6 dB = 4.93; 9 dB = 3.97; 14 dB = 2.82; 18.5 dB = 2.07. At the highest gain setting, three listeners reported ratings of 0, i.e., no muffling.

These data show that changes in signal processing can improve sound quality. The next step will be to implement the gains in a clinical processor and test in real-world environments. This aspect of translational research will take some time. For example, at a minimum, the following issues need to be addressed: (i) where should gains be implemented in the signal processing chain so that existing functions are not altered, (ii) will the implementation negatively impact standard measures of implant performance, (iii) will the regulatory burden be excessive?

## Limitations

The experiments described provide an insight into CI sound quality for patients who are SSD with a contralateral, normal-hearing ear. Patients who are bilaterally deaf may experience a different sound quality because they lack an immediate reference to the sound of a clean signal.

## Final thoughts

Over a decade ago, our goal was to “listen in” on what a CI sounds like. As evidenced in the Supplementary material audio and video files included in this review, we have been modestly successful in doing that. Earlier in this review, we noted the possibility that we would not be successful at all because the perceptual effects of electrical stimulation could not be replicated by acoustic stimulation. This does not seem to be the case. Many patients have created acoustic sound-quality matches that were very close to the sound of their CI. It is not clear whether the very close matches fail to be complete matches because (i) there are, indeed, aspects of electric stimulation that cannot be replicated by acoustic stimulation or (ii) we did not adjust the acoustic stimulation optimally.

Vocoder stimulation did not prove useful in achieving close matches to CI sound quality. This was surprising given the value of vocoders in assessing signal processing options for CIs. However, these studies focused on speech understanding and not on voice quality. Our work indicates that achieving a very close match to CI sound quality likely requires a good approximation to the effects of a normal voice-source interacting with the vocal tract transfer function. If the source is abnormal, as in a sine vocoder, that source might specify the ‘demented robot’ heard by the patient in Supplementary material 3: First patient match.

We have found that band limiting, some amount of spectral smearing, and pitch changes via contour shaping, F0 shifts, or formant shifts, are the predominant acoustic manipulations that yield matches with high similarity ratings to CI sound quality. By listening to the matches in the Supplementary material, the variability in sound quality experienced by CI listeners and the possibilities for improvement with changes in technology can be appreciated. Improvement is crucial because sound quality accounts for cochlear implant-related quality of life outcomes; speech recognition does not (Berg et al., 2025).
